# Increased Prevalence of Severe Fever with Thrombocytopenia Syndrome in Eastern China Clustered with Multiple Genotypes and Reasserted Virus during 2010–2015

**DOI:** 10.1038/s41598-017-06853-1

**Published:** 2017-07-26

**Authors:** Zhifeng Li, Jianli Hu, Lunbiao Cui, Ye Hong, Jianwei Liu, Pengfei Li, Xiling Guo, Wendong Liu, Xiaochen Wang, Xian Qi, Bin Wu, Zhi Feng, Aihua Shen, Xuejian Liu, Hongjun Zhao, Wenwen Tan, Jiangang Zhou, Zheng Xing, Changjun Bao

**Affiliations:** 1 Jiangsu Provincial Center for Disease Prevention and Control, Nanjing, China; 2 Medical School, Nanjing University, Nanjing, China; 3 School of Public Health, Shandong University, Jinan, China; 4 Jiangning Center for Disease Prevention and Control, Jiangning, China; 5 Lishui Center for Disease Prevention and Control, Lishui, China; 6 Xuyi Center for Disease Prevention and Control, Xuyi, China; 7 Yixing Center for Disease Prevention and Control, Yixing, China; 8 College of Veterinary Medicine, University of Minnesota at Twin Cities, Saint Paul, MN USA; 9 Clinical Laboratory, Jiangsu Province Hospital of TCM, Affiliated Hospital of Nanjing University of TCM, Nanjing, China

## Abstract

Severe fever with thrombocytopenia syndrome (SFTS) is a novel tick-borne viral disease with high mortality. Since January 2010, we have conducted an epidemiological surveillance and etiological study of SFTS in Jiangsu and Anhui provinces. From January 2010 through December 2015, a total of 286 SFTS cases were confirmed in Jiangsu and Anhui provinces with a case fatality rate of 16.1%. The majority of confirmed SFTS cases were distributed in the border area of Jiangsu and Anhui provinces. Our findings suggest that the SFTS prevalence rate rose since 2010 and reached its highest in 2015. Phylogenetic analysis demonstrated that the majority of the SFTSV strains (83.6%) from Jiangsu and Anhui provinces belonged to genotypes A and D. Notably, we identified three strains of SFTSV clustered into the genotype E. This is the first report of the genotype E SFTSV strains in mainland of China. A reassortment between genotype A and D was found in the central region of the endemic areas, where three SFTSV genotypes (A, C and D) were co-circulating.

## Introduction

Severe fever with thrombocytopenia syndrome (SFTS) is an emerging infectious disease with a high case fatality rate. The disease is characterized by high fever and a drastic reduction of platelets and leukocytes resulting in multi-organ failure^1^. The case fatality rates reported have varied from 2.5 to 24% depending on the regions where the cases occurred^2–5^. A novel bunyavirus called Severe Fever with Thrombocytopenia Syndrome Virus (SFTSV), a member of *Phlebovirus* genera, is considered the cause of this deadly infection. Currently, there is as yet no vaccine for prevention and specific antiviral therapy for SFTS is lacking clinically^6^.

SFTSV has been thought to be transmitted by ticks, as about 10% of SFTS cases had evident experience of tick bite before onset of the disease and the virus was detected or isolated from some species of ticks, such as *Haemaphysalis longicornis*, *Amblyommate studinarium*, and *Ixode snipponensis* ^7–9^. Previous studies conducted in Jiangsu and Shandong provinces of China showed that many domestic animals (e.g., goats, cows, and dogs) and small wild animals (e.g., *Sorex araneus*, *Erinaceus europaeus*, *Mus musculus*, and *Suncus murinus*) could be infected by SFTSV with no or only inconspicuous symptoms, which may likely serve as amplifying hosts of SFTSV in nature^9–13^.

SFTSV is prevalent mainly in seven central-eastern provinces of China including Henan, Hubei, Anhui, Jiangsu, Zhejiang, Shandong, and Liaoning with over 50 percent of reported SFTS cases occurring around Mount Dabieshan located in the border area of Henan and Hubei provinces^1^. All of laboratory-confirmed SFTS cases occurred during the period from March to December, with epidemic peaking from May to July, and most of the patients were farmers living in hilly areas^14–17^. Since 2012 SFTS infections have been also reported in South Korea and Japan^3, 4^. Bayesian analysis based on the SFTSV single genomic segment suggested that SFTSV was likely originated in Dabieshan areas 50–225 years ago and the isolated SFTSV stains so far have been classified into five genotypes (genotype A-E)^18^. The majority of the SFTSV strains from mainland of China belong to genotype A, B and D, while most of the isolates from South Korea, Japan, and Zhoushan Islands of Zhejiang Province, China, fall into the genotype E based on different phylogeographic analysis, suggesting a possible cross-ocean transmission of the genotype E virus^17–20^. Our recent study showed that some species of migratory birds, such as *Anser cygnoides* and *Streptopelia chinensis*, could be parasitized by *Haemaphysalis longicornis* and infected by SFTSV, which might contribute to a long-distance spread of SFTSV via migratory flyways^9^. The eastern China including Jiangsu and Zhejiang provinces is adjacent to Anhui and the Dabieshan area, and Jiangsu, like Zhejiang, is also a coastal province across Eastern China Sea from South Korea and Japan. These two provinces are also situated along the migratory routes of different bird species from Zhoushan Islands in Zhejiang to South Korea or Japan. Thus, the eastern China is the critical area where migratory birds-borne transmission or even reassortants of different SFTSV genotypes could happen. In this study we have conducted a comprehensive survey of SFTS in Jiangsu and Anhui provinces with data collected in six consecutive years from 2010 to 2015 to elucidate the epidemiological and molecular features of SFTSV. Our results showed that multiple genotypes of SFTSV are co-circulating in Jiangsu and Anhui provinces and a reassortment between genotype A and D happened in these areas. We also identified three SFTSV strains of genotype E, which was previously reported to circulate only in Japan, the Korean peninsula, and Zhoushan Islands of Zhejiang Province.

## Results

### Increased SFTS cases and Seasonality of SFTSV infection during 2010-2015

**Figure 1:**
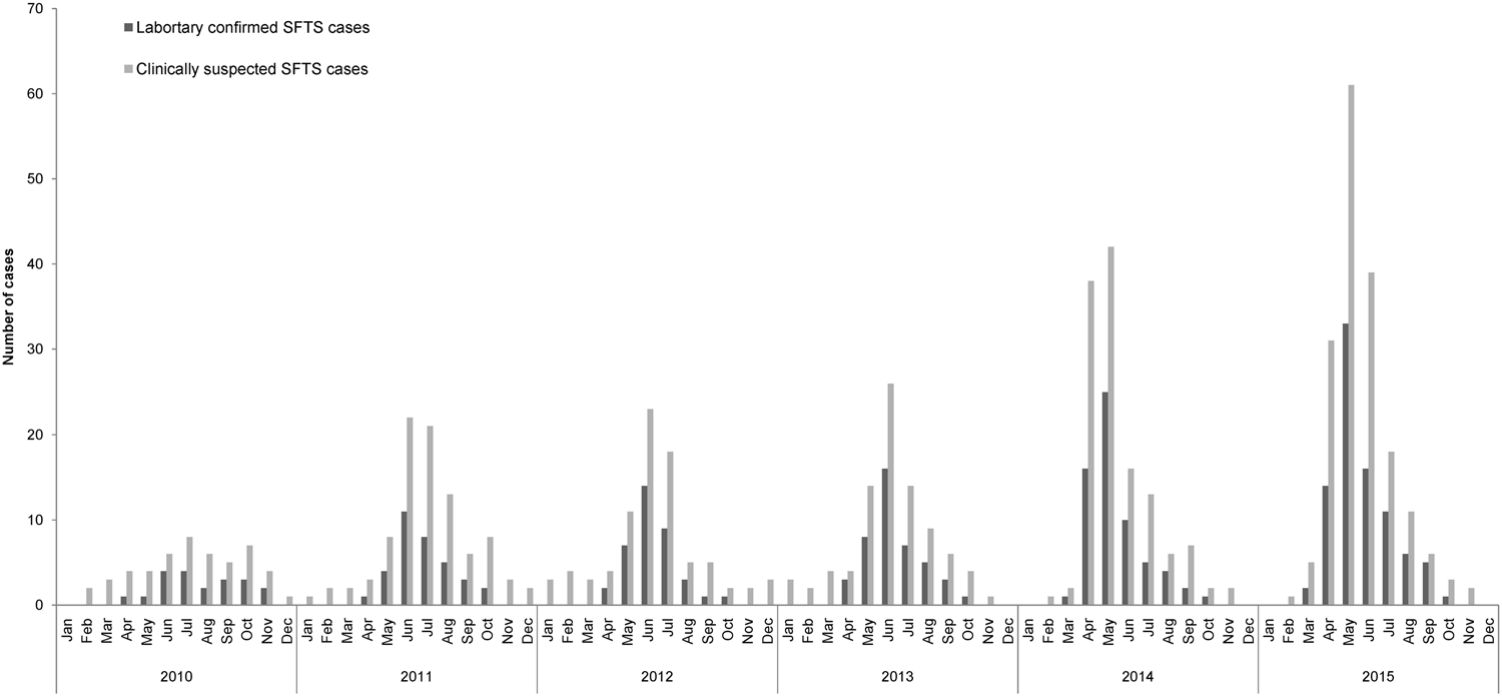
Epidemiological curve showing the seasonality of SFTS cases in Jiangsu and Anhui province during 2010 and 2015.

In Jiangsu and Anhui provinces SFTS was initially identified around 2009, starting with sporadic cases. Outbreaks of SFTS occurred from 2010, and in 2014 and 2015 there were more cases reported than in earlier years, and the case number in 2015 increased by 435%, 249% and 229% compared to that in 2010, 2011, and 2012, indicating that SFTSV spread rapidly and endemic areas expanded (Fig. 1). On the other hand, case fatality rate of SFTS varied. It was up to 35% in 2010, but decreased to 5% in 2012. However, the deaths rate was kept increasing since 2013 and reached to 17.2% in 2015.

**Figure 2:**
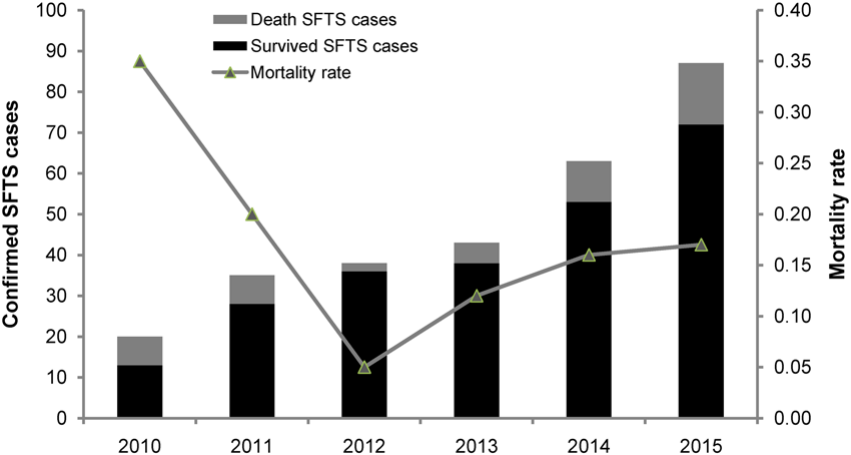
The mortality of SFTS cases in Jiangsu and Anhui province during 2010 and 2015.

All of laboratory-confirmed SFTS cases occurred during March to November, with epidemic peaking from May to July (65.4%). In 2010–2013, case number peaked in June (41.2%), while in 2014 and 2015, the peak occurred in May (47.6%) (Fig. 2). There are two features worth of mentioning for monthly distribution of the cases: 1) the peak of monthly cases advanced by one month from June during 2010–2013 to May during 2014–2015; 2) the first batch of confirmed cases appeared earlier in March during 2014–2015 than April during 2010–2013.

### Demographic profile and clinical manifestation

A total of 647 SFTS-like cases were reported from January 2010 to December 2015. Among them 215(33.2%) cases were positive for SFTSV-specific RNA and 187(28.9%) cases were positive for the viral IgM antibodies, detected in patients’ acute phase sera. Combined, 289(44.7%) cases were positive by these two methods. All the SFTSV-specific RNA positive patients were laboratory confirmed by virus isolation or a four-fold elevation of the IgG antibodies against SFTSV in paired serum samples by IFA. Three patients, with SFTSV-specific IgM antibodies but without SFTSV-specific RNA in the acute phase sera were out-ruled. So, a total of 286 patients were laboratory confirmed by SFTSV infection.

Among the 286 confirmed SFTS patients who had IgM and IgG tested in the acute phase sera, the median interval between onset of illness and positive result in the initial SFTSV specific IgM test was 8.5 days (range, 2–18 days). A total of 79 SFTS patients had a positive result of IgG antibodies in the acute phase sera, and the median interval between onset of illness and positive result was 11.5 days (range, 7–19 days). Forty-six (16.1%) of the confirmed SFTS patients died, and the median duration from the onset of symptoms until death was 10.5 days (range, 2–33 days).

Approximately 47.8% of the confirmed SFTS patients were male and 52.2% were female (1:1.09 ratio), and the difference was not statistically significant (*P* = 0.54) (Table 1).

**Table 1:** Demographics and clinical features of confirmed and ruled-out cases with SFTSV infection in Eastern China, 2010–2015.

Characteristics	All Clinical SFTS Cases		*P* value	Confirmed SFTS Cases		*P* value
	Confirmed SFTS Cases (N = 286)	Ruled-out Cases (N = 324)		Fatal (N = 46)	Not-Fatal (N = 240)	
**Demographics No. (%)**						
Age (years)						
<30	2(0.7)	24(7.4)	<**0.001**	0(0)	2(0.8)	0.53
30–60	135(47.2)	193(59.6)	**0.002**	8(17.4)	127(52.9)	<**0.001**
>60	149(52.1)	107(33.0)	<**0.001**	38(82.6)	111(46.3)	<**0.001**
Sex						
Male	142(49.6)	169(52.2)	0.54	22(47.8)	122(50.8)	0.71
Female	144(50.4)	155(47.8)	0.54	24(52.2)	120(49.2)	0.79
Marital status						
Married	236(82.5)	260(80.2)	0.47	40(87.0)	196(81.7)	0.39
Not married	50(17.5)	64(19.8)	0.47	6(13.0)	44(18.3)	0.39
Occupation						
Farming	253(88.5)	212(65.4)	<**0.001**	42(91.3)	211(87.9)	0.51
Non-farming	33(11.5)	112(34.6)	<**0.001**	4(8.7)	29(12.1)	0.51
Tick bites	75(26.2)	26(8.0)	<**0.001**	10(21.7)	65(27.1)	0.45
**Clinical features No. (%)**						
Fever	286(100.0)	324(100.0)	—	46(100)	240(100)	—
Chilly	165(57.7)	162(50.0)	0.07	35(54.3)	130(54.2)	0.42
Fatigue	136(47.6)	130(40.1)	0.08	23(50.0)	113(47.1)	0.87
Headache	143(50.0)	149(46.0)	0.32	28(60.9)	115(47.9)	0.11
Myalgia	168(58.7)	187(57.7)	0.88	29(63.0)	139(57.9)	0.74
Nephralgia	28(9.8)	33(10.2)	0.87	6(13.0)	22(9.2)	0.42
Lymphadenopathy	61(21.3)	20(6.2)	<**0.001**	10(21.7)	51(21.3)	0.83
Anorexia	232(81.1)	268(82.7)	0.61	43(93.5)	209(78.8)	0.22
Nausea	198(69.2)	201(62.0)	0.06	31(67.4)	167(69.6)	0.77
Vomiting	122(42.7)	136(42.0)	0.86	22(47.8)	100(41.7)	0.44
Abdominal pain	78(27.2)	92(28.4)	0.76	14(30.4)	64(26.7)	0.86
Abdominal distension	65(22.7)	76(23.5)	0.80	12(26.1)	53(22.1)	0.55
Diarrhea	155(54.2)	109(33.6)	<**0.001**	26(56.5)	129(53.8)	0.73
Conjunctival congestion	43(15.0)	19(6.6)	<**0.001**	30(65.2)	13(5.4)	<**0.001**
Skin petechiae	56(19.6)	41(12.7)	**0.02**	31(67.4)	25(10.4)	<**0.001**
Gum bleed	49(17.1)	31(9.6)	**0.01**	31(67.4)	18(7.5)	<**0.001**
Haematemesis	36(12.6)	21(6.3)	**0.01**	27(58.7)	19(7.9)	<**0.001**
Leukopenia	216(75.5)	56(17.3)	<**0.001**	41(89.1)	175(72.9)	**0.02**
Thrombocytopenia	191(66.8)	126(38.9)	<**0.001**	45(97.8)	146(60.8)	<**0.001**

For individuals in which age information was available, we stratified them into three different age groups. Those over 60 years represented 52.1% of all confirmed SFTS patients, who also constituted the majority (82.6%) of all fatal cases (*P* < 0.001) while no age difference was detected for non-fatal cases (46.3% vs. 52.9%) (Table 1). Most confirmed SFTS patients were farmers (88.5%), and 26.2% of clinical SFTS cases were bitten by ticks and confirmed to be SFTSV infection, while only 8% of the ruled-out cases were bitten by ticks (*P* < 0.001) (Table 1). The median incubation period of confirmed cases was 8.5 days, ranging from 1 day to 34 days according to the patients with definite tick bite experience before onset of illness.

Most of the fatal cases were shown to have conjunctival congestion (65.2%), skin petechiae (67.4%), and gum bleed (67.4%), and up to 89.1% and 97.8% of the fatal patients had leukopenia and thrombocytopenia, respectively (Table 1), which may play a vital role in viral pathogenesis in human SFTSV infection

### Geographical distribution of SFTS cases

As for 647 SFTS-like cases reported from January 2010 to December 2015 in Jiangsu and Anhui provinces, the vast majority of confirmed SFTS cases (73.2%) were distributed in the border area of the two provinces, which was a long and narrow area (Fig. 2). There were two parts of the epidemic area with SFTS cases relatively concentrated. One part (57.0%) located in the north of the epidemic area, which includes Xuyi, Mingguang, Nanqiao, Laian and Diongyuan counties. The other part (16.2%) including Lishui, Liyang and Yixing, was in the south of the epidemic area.

### Multiple genotypes mixed in the endemic areas of the eastern China

**Figure 3:**
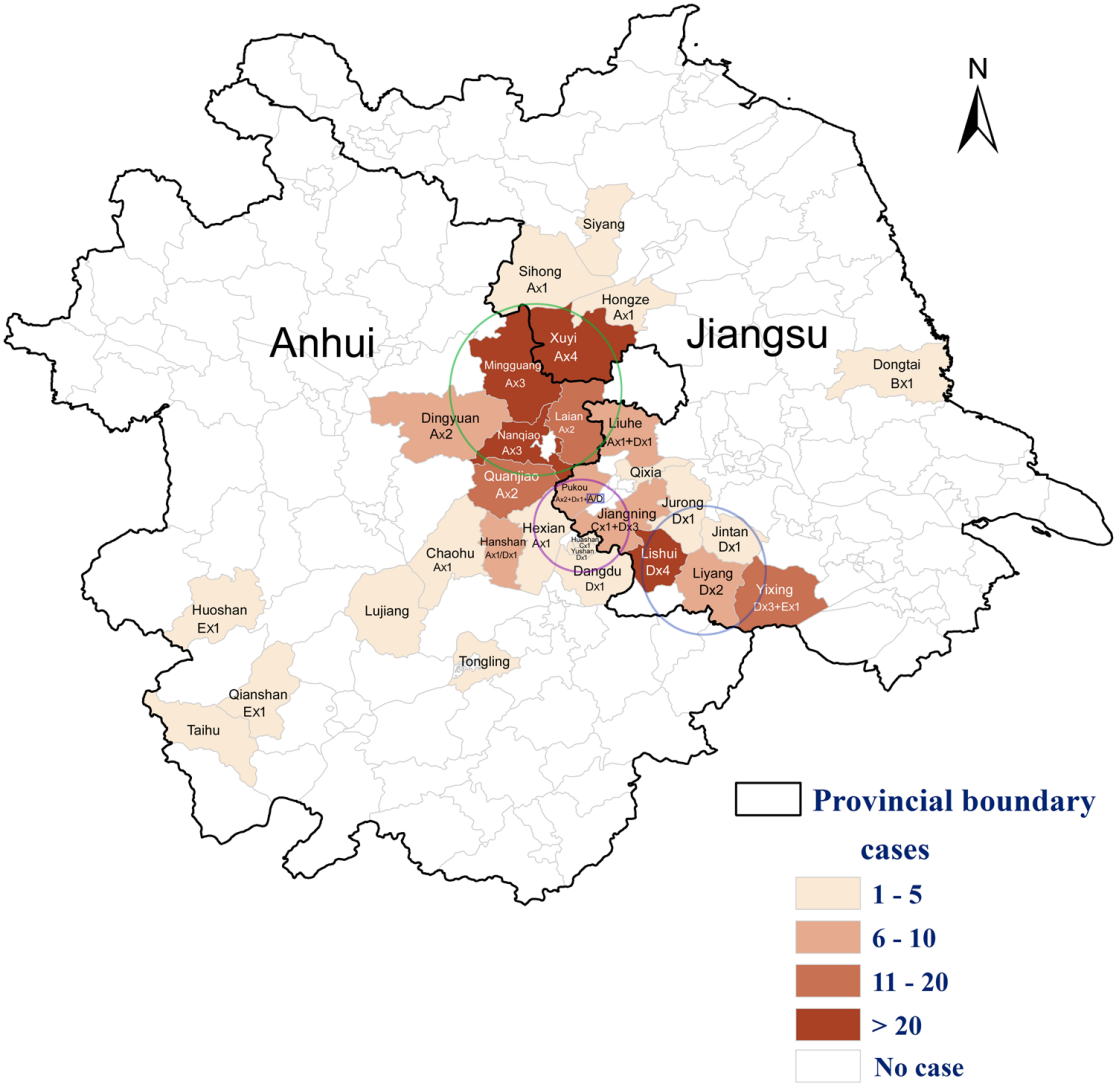
The geographical distribution of SFTS cases and SFTSV genotypes in Jiangsu and Anhui provinces. This map was created using the software ArcGIS version 10.0 (http://www.esri.com/). A, C, D, and E represented genotype A, C, D, and E of SFTSV strains distributed in each county of the SFTS endemic areas; A/D represented reassortment between SFTSV genotype A and genotype D; The green, purple and blue circles represented northern, central and southern part of the endemic areas in study, respectively.

**Figure 4:**
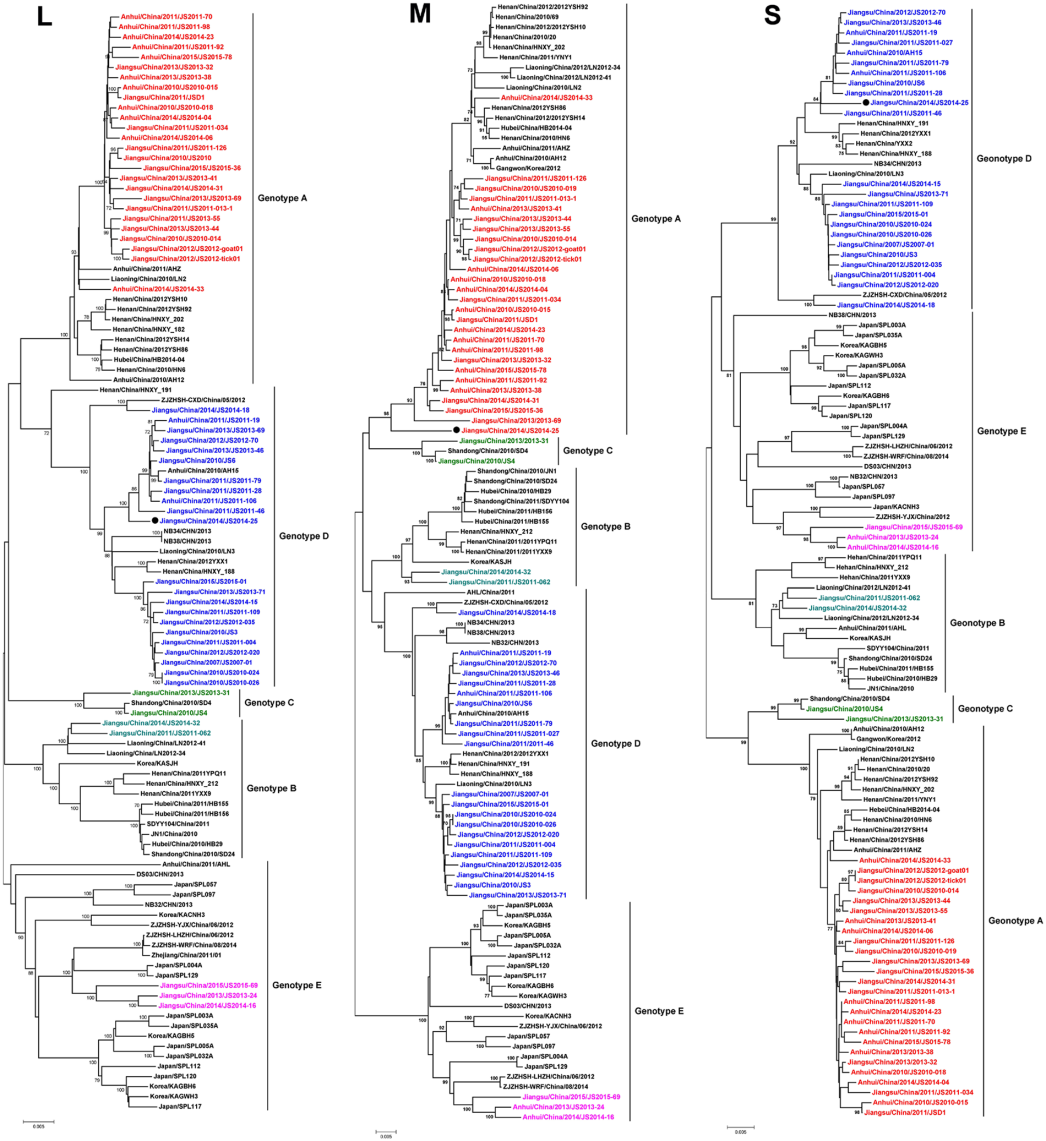
Phylogenetic analysis of SFTSV strains isolated from Jiangsu and Anhui provinces during 2010 and 2015, compared with SFTSV strains from other areas. The phylogenetic trees were constructed using the Maximum Likelihood Method with the MEGA5.1 software based on L, M and S segments of SFTSV strains from different SFTS endemic areas. The reliability values indicated at the branch nodes were determined using 1,000 bootstrap replications. Bootstrap value ≥70 were labeled at nodes. The sequences were named according to province/country/year of collection or isolation/strain. Colored taxon names of the phylogenetic trees presented SFTSV strains isolated in this study. The red taxon names were the SFTSV strains of Jiangsu and Anhui provinces distributed in genotype A, while green, cyan, blue and pink represented SFTSV strains of this study clustered in genotype B, C, D and E, respectively. The SFTSV strains isolated from north part of the epidemic area of Jiangsu and Anhui provinces distributed into genotype A and most of the SFTSV strains from south part of the epidemic area belong to genotype D. There were two SFTSV strains from the central of the epidemic areas clustered into genotype C. There were three SFTSV strains, two from Huoshan and Qianshan of Anhui and one from Yixing of Jiangsu, clustered into genotype E. The SFTSV strain with reassortment was labeled with black solid squares.

Only SFTSV strains with complete genome sequences were analyzed in this study. Among the 110 SFTSV strains selected, 55 strains were from this study. All SFTSV strains were isolated between 2007 and 2015. Phylogenetic analysis classified all SFTSV strains into 5 lineages in each segment, named genotype A, B, C, D and E (Fig. 3). The SFTSV strains isolated from the northern part of the epidemic area in Jiangsu and Anhui province distributed into genotype A and most of the SFTSV strains from the southern part of the epidemic area belonged to genotype D. There were two SFTSV strains (JS2014–32 and JS2011-62) from the central of the endemic areas clustered into genotype C (Fig. 4).

Interestingly, in our study we found that there were three SFTSV isolates, two from Huoshan and Qianshan of Anhui (JS2013-24 and JS2014-16) and one Yixing from Jiangsu (JS2015-69), clustered into genotype E (Fig. 4), which has isolates originated from South Korea^14^, Japan, and a dozen of isolates from Zhoushan Islands of Zhejiang province.

Multiple genotypes of the SFTSV clustered in this area, suggesting a possibility that genomic reassortment could occur, which, however, has not been reported in this endemic region. We performed a reassortment analysis among SFTSV strains from this study and found an SFTSV strain (Jiangsu/China/2014/JS2014-25) from Pukou district of Jiangsu Province which is composed of the L and S segments of genotype D, and the M segment of genotype A (Fig. 4). The reassortment event in M segment was identified by RDP, GENECONV, Bootscan, SiScan, and 3Seq methods and supported by significant p-values of 1.25E-11.

## Discussion

In this study, we described the epidemiological and etiological characteristics of SFTS in eastern China including Jiangsu and Anhui provinces, in six consecutive years, from January 2010 to December 2015. A distinct seasonality with epidemic peaking from May to July for SFTSV infection in this area was observed, which was identical to other SFTS epidemic areas, such as those in Henan, Hebei, Shandong, and Zhejiang provinces [14–16]. The number of cases peaked during the summer season, probably due to increased tick density. *Haemaphysalis longicornis* is proved to be the vector of SFTSV transmission. Studies have shown a correlation between tick densities and SFTS incidence among human beings^21^. Most confirmed SFTS cases in this study distributed in the border areas of Jiangsu and Anhui provinces, which are hilly areas with intensive tick activity in summer season. Over 80% of the SFTS cases were farmers who had higher probability of getting bitten by ticks when undertaking farm work.

The case fatality rate of SFTS varied. It was up to 35% in 2010, but decreased to 5% in 2012. Improved clinical care and early diagnosis could help reduce the case fatality rates. However, the deaths rate was kept increasing since 2013 and reached to 17.2% in 2015. Changed virulence of viral strains in the endemic areas or variable genetic resistance in the population of new endemic areas may also influence the case fatality rates. We have not detected apparent gene mutations to demonstrate the increase of viral virulence from the field isolates recent years. The data of genetic resistance to SFTSV in the population of Jiangsu and Anhui provinces are not available at present. So, our current knowledge could not explain the increasing case fatality rates.

Our study also showed increased SFTS cases in 2014 and 2015 compared with 2010–2013.We have also identified that the peak incidence of monthly cases had advanced from June to May (Fig. 1) during 2014–2015, which was one month later during 2010–2013. The earlier appearance of the cases recent years may be explained by wider spread and transmission of the virus and increased contact of humans with virally infected ticks. But our current knowledge could not explain the peak shift. Detailed survey of tick densities, seasonal fluctuation dynamics and infection rates of SFTSV should be further conducted in order to understand the endemic features and develop more effective measure to prevent the rapid spread of this deadly virus.

Most primary clinical manifestations of SFTSV infection were non-specific, only 44.2% of the clinically suspected SFTS cases, diagnosed by clinicians according to the national clinical guidelines, were eventually confirmed. Our results showed that diarrhea, superficial lymphadenopathy, and bleeding tendency were more common in confirmed SFTS cases compared with ruled-out ones (P < 0.01) (Table 1). These clinical features could be helpful to clinicians in making clinical diagnosis of SFTSV infection. Up to 89.5% and 97% of the fatal cases had lymphopenia and thrombocytopenia, respectively, which underscores the tropism of hemopoietic cells in the SFTSV pathogenesis and disease development. Drastic loss of platelets leads to internal bleeding and some symptoms. More importantly, loss of platelets and white blood cells means that these cells disrupt and die, due to apoptosis or other reasons. The debris of platelets and leukocytes are going to aggregate in and clot the microvessels in organs and tissues, which could contribute to failure of multiple organs, the direct cause of death in SFTS patients^22^.

Previous studies showed that old age was a risk factor for SFTSV infection^23^. Our results demonstrated that 52.4% of the confirmed SFTS cases were individuals over 60 years old in eastern China, consistent with the data from the earlier studies in other SFTS-endemic areas^19, 23^. Likewise, SFTSV were more pathogenic to older people, as over 80% of the fatal SFTS cases were the population over 60 years old as shown in our report (Table 1).

We amplified and sequenced SFTSV genomic sequences circulating in Jiangsu and Anhui provinces, and performed phylogenetic analysis with all genomic sequences available in the GenBank about the isolates from the mainland of China, the Zhoushan Islands of Zhejiang Province and other countries including South Korea and Japan. Phylogenies of three genomic segments of SFTSV showed five clades defined accordingly as SFTSV genotypes A-E (Fig. 3). Genotype A, B, C and D are mainly circulated in the mainland of China which is similar to previous findings^18, 20, 24^. The majority of SFTSV strains in Jiangsu and Anhui provinces (83.6%) belong to genotype A and D. Two SFTSV strains named JS4 and JS2013–31, previously considered as SFTSV strains from Jiangsu^18, 20, 24^, were excluded from this study as our epidemic survey showed that the two were from SFTS cases infected in Shandong Province.

In this study we identified that three isolates of SFTSV, two from Huoshan and Qianshan of Anhui and one from Yixing of Jiangsu, were clustered into genotype E (Fig. 3), which was circulating only in Japan, the Korean peninsula and Zhoushan Islands of Zhejiang Province as reported previously^18, 20, 24^. This is the first report of the SFTSV strains from the mainland of China that were clustered into genotype E. Previous studies have indicated that migratory birds could be infected by SFTSV or carry SFTSV-infected ticks, which could play an important role in long-distance transmission spreading SFTSV via migratory flyways^9, 24^. Huoshan and Qianshan are located in the south of Anhui Province, hundreds of kilometers away from Yixing in the south of Jiangsu Province. These regions, however, are all on the migratory bird routes with the Zhoushan islands through Japan and the Korean peninsula^24^. It is therefore likely that the same genotype of SFTSV would be located in regions geographically distal to each other and the virus is transmitted long distance via migratory birds.

We also demonstrated evidence to show that genomic reassorment could occur in the endemic areas in the Eastern China with multiple genotypes co-existed in nature. There were three genotypes of SFTSV (genotype A, C and D) co-circulating in the central part of this SFTSV endemic area in this study (Fig. 3). In this study, we report that a reassortment event happened to an SFTSV strain (JS2014-25) isolated from a fatal patient in Pukou district of Jiangsu Province (Fig. 3). Although the reassortment appears to be rare in our study, it may happen more often in nature as a previous study reported reassortments of seven SFTSV strains from the mailand of China^18^. Reassortment of genomic segments is an important mechanism that increases genetic diversity and virulence of segmented RNA viruses^25, 26^. The case fatality rate of the confirmed SFTS cases in this study was 16.1%, which was significantly higher than the case fatality rate reported in other endemic areas. We do not know if the reasserted viruses, that were present in this area, may be attributed to relatively higher case fatality rates. Further surveillances and study of SFTSV genomic mutation in nature and their associated virulence in eastern China should be performed in order to better understand the pathogenicity and pathogenesis of the virus in humans.

## Materials and Methods

### Ethics statement

All experimental procedures with human samples were in strict accordance with the recommendations in the Guide for the Use of Human Samples and approved by the Medical Research Ethics Committees of Jiangsu Provincial Center for Disease Control and Prevention. Suspected SFTS patients from hospitals and primary health care centers were invited to participate in the study and enrolled after written informed consents were obtained from all participants. For children participants, written informed consents were sought from their parents/legal guardians.

### Specimen and data collection

According to the national guidelines^27^, a clinically suspected case of SFTS was defined as fever or a history of fever lasting 2 to 7 days of unknown causes with two or more of the following: leucopenia, thrombocytopenia, myalgia, diarrhea, lymphadenopathy and hemorrhagic manifestation. Other diseases, including hemorrhagic fever with renal syndrome, human granulocytic anaplasmosis, dengue fever, thrombocytopenic purpura, septicaemia were ruled out. A laboratory-confirmed case of SFTS met at least one of the following criteria: 1) isolation of SFTSV by cell culture; 2) a positive result for SFTSV RNA by real-time RT-PCR; 3) a positive result for anti-SFTSV IgM antibody by enzyme-linked immunosorbent assay (ELISA); 4) seroconversion or a four-fold elevation of IgG antibodies against SFTSV by ELISA or indirect immunofluorescence assay (IFA).

Structured questionnaires were conducted by trained staff. The following data were collected: demographic information (age, sex, occupation, and residential or working addresses); clinical data (onset date of symptoms, clinical symptoms, laboratory findings); a tick exposure; presence of any underlying disease (Asthma, chronic bronchitis emphysema, chronic kidney disease etc.). Each case was geo-referenced to a digital map of Jiangsu an Anhui provinces according to his or her residential addresses.

### Viral RNA extraction and detection

Acute phase blood samples were collected from the suspected SFTS patients. Sera were separated from the blood samples by centrifugation at 3000 rpm for 10 min at 4 °C. Serum samples were stored at −80 °C until further use. RNA was extracted from 140 μL serum samples using QIAamp Viral RNA Mini kit (Qiagen, Hilden, Germany) according to the manufacturer’s instructions.

SFTSV RNA in acute-phase sera of SFTS patients were detected with a real-time RT-PCR assay. The real-time RT-PCR assay was performed using primers previously described^28^ and the QuantiTect Probe RT-PCR Kit (Qiagen, Hilden, Germany). The conditions for real-time RT-PCR reaction were as follows: 50 °C for 30 min, 95 °C for 15 min, 40 cycles of 95 °C for 15 s, 60 °C for 1 min. Data were analyzed using the software supplied by the manufacturer.

### SFTSV specific antibodies detection

Acute-phase sera of SFTS patients were detected for anti-SFTSV IgM antibodies using an ELISA kit (Xinlianxin, Wuxi, China) according to the manufacturer’s protocol. Anti-SFTSV IgG antibodies in serum samples were detected as total antibodies including IgG and IgM with an antigen-sandwich ELISA kit (Xinlianxin, Wuxi, China). In initial screening, an undiluted serum sample was used to determine whether the sample was positive for antibodies against SFTSV. Positive serum samples were further diluted in 2-fold increments starting at 1:2 for titration of antibody titers with the same assay.

### Virus Isolation

All viral RNA-positive acute-phase sera of SFTS patients were used to inoculate Vero and DH82 cells for virus isolation as previous described^1^. Briefly, the serum extracts were inoculated onto the Vero cell for two weeks. The culture supernatants were harvested and re-inoculated onto fresh DH82 cells. Cytopathic effect (CPE) on inoculated DH82 cells was examined daily under a light microscope. Isolated viruses were further identified by real-time RT-PCR.

### Immunofluorescence Assay (IFA)

SFTSV-specific IgG antibodies were detected in all human sera by IFA as previously described^29^. Twenty microliters of diluted (1:2 to 1:1280) serum samples were added to the cell-spotted coverslips with viral antigens and incubated for 45 min at 37 °C. After washing, 20 μL of FITC-conjugated goat anti-human IgG (Abcam, UK) diluted 1:80 with Phosphate Buffered Saline (PBS) containing Evans Blue (1:20,000) was added for further incubation for 30 min at 37 °C. After three washes, the slides were mounted in glycerin and examined under an immunofluorescence microscope.

### SFTSV Genome Sequencing

Whole genome sequences of SFTSV strains isolated from SFTS cases (Table S1) were amplified by RT-PCR using the primers described in previous studies^30^. The products were sent to Sangon Biotech (Shanghai, China) for Sanger DNA sequencing. The termini of viral RNA segments were determined with the First Choice RLM-RACE Kit (Invitrogen, USA).

### Phylogenetic analysis

All available SFTSV sequences were downloaded from GenBank database including L, M, and S sequences of SFTSV as references. In total, 330 sequences from 110 strains included 55 strains from Jiangsu and Anhui provinces and 55 strains from other areas were obtained for phylogenetic analysis (See Supplementary Table S1). Some closely related sequences from the same area were not included. Sequences generated from this study and obtained from GenBank were divided into L, M, and S datasets for separate phylogenetic analysis. The sequences in each dataset were aligned using the multiple alignment program ClustalX in BioEdit (version 7.1.3.0) software (Ibis Biosciences, Carlsbad, California, USA). Phylogenetic trees were generated using the Maximum Likelihood Method in the Molecular Evolutionary Genetics Analyses (MEGA) software, version 5.10. Bootstrap probabilities for 1,000 iterations were calculated to evaluate confidence estimates.

### Reassortment analysis

Prior to reassortment analyses, all sequences from Anhui and Jiangsu provinces were aligned using the multiple alignment program Clustal X. To prevent potential biases during phylogenetic inference due to recombination, the concatenated sequences were analyzed with Recombination Detection Program version 3.44 (RDP3) that incorporates RDP, GENECONV, Bootscan, Maxchi, Chimaera, SiScan and 3Seq methods^31, 32^ to uncover evidence for recombination events. Only events with p-values ≦ 0.05 which were detected by three or more methods were considered, employing the Bonferroni correction to avoid false positive results.

### Statistical analysis

EpiData 3.1 was used to construct database. Data were inputted by two individuals. Statistical analysis was performed using SPSS Statistics software version 16.0 (SPSS Inc., Chicago, IL) Pearson chi-square test was used to analyze demographic factors and clinical manifestations of SFTS patients. A probability value of p < 0.05 was considered statistically significant.

### Disclaimers

The opinions expressed by authors contributing to this journal do not necessarily reflect the opinions of the Centers for Disease Control and Prevention or the institutions with which the authors are affiliated.

## Electronic supplementary material

Supplementary Information 
