# Supplementary material for: Increased Prevalence of Severe Fever with Thrombocytopenia Syndrome in Eastern China Clustered with Multiple Genotypes and Reasserted Virus during 2010–2015
Source: Sci Rep. 2017 Jul 26;7:6503. doi: 10.1038/s41598-017-06853-1 (PMC5529542; doi:10.1038/s41598-017-06853-1)
Supplement: Supplementary file 1 — Supplementary Information [file 41598_2017_6853_MOESM1_ESM.doc]

Supplementary information

Increased Prevalence of Severe Fever with Thrombocytopenia Syndrome in Eastern China Clustered with Multiple Genotypes and Reasserted Virus during 2010-2015

Zhifeng Li, Jianli Hu, Lunbiao Cui, Ye Hong, Jianwei Liu, Pengfei Li, Xiling Guo, Wendong Liu, Xiaochen Wang, Xian Qi, Bin Wu, Zhi Feng, Aihua Shen, Xuejian Liu, Hongjun Zhao, Wenwen Tan, Jiangang Zhou, Zheng Xing and Changjun Bao

Table S1. The genome sequences of SFTSV strains used in this study

| Sample location | Viral strain | Sample time | Accession number (segment) | | | Source |
| --- | --- | --- | --- | --- | --- | --- |
|  |  |  | L | M | S |  |
| Jiangsu | JS2007-01 | 2007 | JF837593 | JF837594 | JF837595 | This study |
| Jiangsu | JS3 | 2010 | HQ141601 | HQ141602 | HQ141603 | This study |
| Shandong | JS4 | 2010 | HQ141604 | HQ141605 | HQ141606 | This study |
| Anhui | JS6 | 2010 | HQ830169 | HQ830170 | HQ830171 | This study |
| Jiangsu | JS2010-14 | 2010 | JQ317169 | JQ317170 | JQ317171 | This study |
| Anhui | JS2010-15 | 2010 | JQ317172 | JQ317173 | JQ317174 | This study |
| Anhui | JS2010-18 | 2010 | JQ317175 | JQ317176 | JQ317177 | This study |
| Jiangsu | JS2010-19 | 2010 | JQ317178 | JQ317179 | JQ317180 | This study |
| Jiangsu | JS2010-24 | 2010 | HQ830163 | HQ830164 | HQ830165 | This study |
| Jiangsu | JS2010-26 | 2010 | HQ830166 | HQ830167 | HQ830168 | This study |
| Jiangsu | JS2012-goat01 | 2010 | KC473537 | KC473538 | KC473539 | This study |
| Jiangsu | JS2012-tick01 | 2010 | KC473540 | KC473541 | KC473542 | This study |
| Jiangsu | JS2011-04 | 2011 | KC505123 | KC505124 | KC505125 | This study |
| Jiangsu | JS2011-13-1 | 2011 | KC505126 | KC505127 | KC505128 | This study |
| Anhui | JS2011-19 | 2011 | KY362295 | KY362343 | KY362319 | This study |
| Anhui | JS2014-25 | 2014 | KY362305 | KY362353 | KY362329 | This study |
| Jiangsu | JS2011-28 | 2011 | KY362296 | KY362344 | KY362320 | This study |
| Anhui | JS2011-46 | 2011 | KY362297 | KY362345 | KY362321 | This study |
| Jiangsu | JS2015-69 | 2011 | KY362308 | KY362356 | KY362332 | This study |
| Anhui | JS2011-70 | 2011 | KY362298 | KY362346 | KY362322 | This study |
| Jiangsu | JS2011-79 | 2011 | KY362299 | KY362347 | KY362323 | This study |
| Anhui | JS2011-92 | 2011 | KY362300 | KY362348 | KY362324 | This study |
| Anhui | JS2011-98 | 2011 | KY362301 | KY362349 | KY362325 | This study |
| Anhui | JS2011-106 | 2011 | KY362294 | KY362342 | KY362318 | This study |
| Jiangsu | JS2015-26 | 2015 | KY362306 | KY362354 | KY362330 | This study |
| Jiangsu | JS2011-27 | 2011 | KC505129 | KC505130 | KC505131 | This study |
| Anhui | JS2011-34 | 2011 | KC505132 | KC505133 | KC505134 | This study |
| Jiangsu | JS2011-62 | 2011 | KC505135 | KC505136 | KC505137 | This study |
| Jiangsu | JS2011-109 | 2011 | KC505138 | KC505139 | KC505140 | This study |
| Jiangsu | JSD1 | 2010 | JF267783 | JF267784 | JF267785 | This study |
| Jiangsu | JS2012-020 | 2012 | KC505141 | AKC505142 | KC505143 | This study |
| Jiangsu | JS2012-035 | 2012 | KC505144 | KC505145 | KC505146 | This study |
| Anhui | JS2012-70 | 2012 | KY362302 | KY362350 | KY362326 | This study |
| Anhui | JS2013-24 | 2013 | KY362310 | KY362358 | KY362334 | This study |
| Jiangsu | JS2013-31 | 2013 | KY362303 | KY362351 | KY362327 | This study |
| Anhui | JS2013-32 | 2013 | KY362311 | KY362358 | KY362335 | This study |
| Anhui | JS2013-38 | 2013 | KY362312 | KY362360 | KY362336 | This study |
| Anhui | JS2013-41 | 2013 | KY362313 | KY362361 | KY362337 | This study |
| Jiangsu | JS2013-44 | 2013 | JQ317229 | JQ317230 | JQ317231 | This study |
| Jiangsu | JS2013-46 | 2013 | JQ317232 | JQ317233 | JQ317234 | This study |
| Jiangsu | JS2013-55 | 2013 | KY362304 | KY362352 | KY362328 | This study |
| Anhui | JS2013-69 | 2013 | JQ317238 | JQ317239 | JQ317240 | This study |
| Jiangsu | JS2013-71 | 2013 | JQ317241 | JQ317242 | JQ317243 | This study |
| Anhui | JS2014-04 | 2014 | KY362314 | KY362362 | KY362338 | This study |
| Anhui | JS2014-06 | 2014 | KY362315 | KY362363 | KY362339 | This study |
| Jiangsu | JS2014-15 | 2014 | JQ317250 | JQ317251 | JQ317252 | This study |
| Anhui | JS2014-16 | 2014 | JQ317253 | JQ317254 | JQ317255 | This study |
| Jiangsu | JS2014-18 | 2014 | JQ317256 | JQ317257 | JQ317258 | This study |
| Anhui | JS2014-23 | 2014 | JQ317259 | JQ317260 | JQ317261 | This study |
| Jiangsu | JS2014-31 | 2014 | JQ317262 | JQ317263 | JQ317264 | This study |
| Anhui | JS2015-32 | 2015 | KY362317 | KY362365 | KY362341 | This study |
| Jiangsu | JS2014-33 | 2014 | JQ317268 | JQ317269 | JQ317270 | This study |
| Jiangsu | JS2015-36 | 2015 | KY362307 | KY362355 | KY362331 | This study |
| Anhui | JS2015-78 | 2015 | KY362309 | KY362357 | KY362333 | This study |
| Jiangsu | JS2015-01 | 2015 | KY362316 | KY362364 | KY362340 | This study |
| Anhui | AH12_China_2010 | 2010 | HQ141591 | HQ141590 | HQ141589 | Genbank |
| Anhui | AH15_China_2010 | 2010 | HQ141592 | HQ141593 | HQ141594 | Genbank |
| Anhui | Anhui/China/2011/AHL | 2011 | JQ670934 | JQ670930 | JQ670932 | Genbank |
| Anhui | AHZ_China_2011 | 2011 | JQ670929 | JQ670930 | JQ670931 | Genbank |
| Henan | 2011YGS5 | 2011 | KF711884 | KF711925 | KF711893 | Genbank |
| Henan | 2011YGS7 | 2011 | KF711885 | KF711922 | KF711895 | Genbank |
| Henan | 2011YPQ11 | 2011 | KF711886 | KF711926 | KF711897 | Genbank |
| Henan | 2011YPQ17 | 2011 | KF711887 | KF711923 | KF711896 | Genbank |
| Henan | 2011YSC22 | 2011 | KF711888 | KF711927 | KF711894 | Genbank |
| Henan | 2011YSC60 | 2011 | KF711863 | KF711945 | KF711899 | Genbank |
| Henan | 2011YXX9 | 2011 | KF711889 | KF711924 | KF711898 | Genbank |
| Henan | 2012YGS10 | 2012 | KF711872 | KF711937 | KF711909 | Genbank |
| Henan | 2012YSH14 | 2012 | KF711876 | KF711938 | KF711910 | Genbank |
| Henan | 2012YSH86 | 2012 | KF711877 | KF711939 | KF711907 | Genbank |
| Henan | 2012YSH89 | 2012 | KF711873 | KF711935 | KF711906 | Genbank |
| Henan | 2012YSH92 | 2012 | KF711865 | KF711941 | KF711917 | Genbank |
| Henan | 2012YSH93 | 2012 | KF711866 | KF711946 | KF711913 | Genbank |
| Henan | 2012YSH104 | 2012 | KF711867 | KF711943 | KF711912 | Genbank |
| Korea | Gangwon_Korea_2012 | 2012 | KF358691 | KF358692 | KF358693 | Genbank |
| Hebei | HB155_China_2011 | 2011 | JQ733564 | JQ733563 | JQ733565 | Genbank |
| Hebei | HB156_China_2011 | 2011 | JQ733567 | JQ733566 | JQ733568 | Genbank |
| Henan | Henan_CHN isolate 69 | 2010 | JF682776 | JF682777 | JF682778 | Genbank |
| Henan | HN6_China_2010 | 2010 | HQ141595 | HQ141596 | HQ141597 | Genbank |
| Henan | HN13_China_2010 | 2010 | HQ141598 | HQ141599 | HQ141600 | Genbank |
| Henan | HN-YNY1 | 2011.6 | KF356552 | KF356538 | KF356526 | Genbank |
| Henan | HN-YPQX03 | 2011 | KF356550 | KF356539 | KF356527 | Genbank |
| Henan | HN-YSHX002 | N | KF356551 | KF356540 | KF356528 | Genbank |
| Henan | 2010-FQM | 2010 | HQ419227 | HQ419236 | HQ419240 | Genbank |
| Japan | JPPL003A | 2012 | AB817980 | AB817988 | AB817996 | Genbank |
| Japan | JPPL004A | 2012 | AB817981 | AB817989 | AB817997 | Genbank |
| Japan | JPPL005A | 2012 | AB817982 | AB817990 | AB817998 | Genbank |
| Japan | JPPL032A | 2012 | AB817985 | AB817993 | AB818001 | Genbank |
| Japan | JPPL035A | 2012 | AB817986 | AB817994 | AB818002 | Genbank |
| Liaoning | LN2_China_2010 | 2010 | HQ141607 | HQ141608 | HQ141609 | Genbank |
| Liaoning | LN3_China_2010 | 2010 | HQ141610 | HQ141611 | HQ141612 | Genbank |
| Liaoning | LN2012-34 | 2012 | KF887442 | KF887437 | KF887432 | Genbank |
| Liaoning | LN2012-41 | 2012 | KF887443 | KF887438 | KF887433 | Genbank |
| Shandong | JN1_China_2010 | 2010 | JN258707 | JN258706 | JN258705 | Genbank |
| Shandong | SD4_China_2010 | 2010 | HM802202 | HM802203 | HM802204 | Genbank |
| Shandong | SD24_China_2010 | 2010 | HM802200 | HM802201 | HM802205 | Genbank |
| Korea | KACNH3 | 2014 | KP663734 | KP663735 | KP663736 | Genbank |
| Korea | KAGBH5 | 2014 | KP663734 | KP663738 | KP663739 | Genbank |
| Korea | KAGBH6 | 2014 | KP663740 | KP7663741 | KP663742 | Genbank |
| Korea | KAGWH3 | 2014 | KP663743 | KP663744 | KP663745 | Genbank |
| Korea | KASJH | 2014 | KP663746 | KP663747 | KP663748 | Genbank |
| Zhejiang | DS02-CHN-2013 | 2013 | KR698345 | KR698332 | KR698319 | Genbank |
| Zhejiang | DS03-CHN-2013 | 2013 | KR698346 | KR698333 | KR698320 | Genbank |
| Henan | HN-LR-China-05-2012 | 2012 | KR017842 | KR017861 | KR017823 | Genbank |
| Zhejiang | NB32-CHN-2013 | 2013 | KR698352 | KR698339 | KR698326 | Genbank |
| Zhejiang | NB34-CHN-2013 | 2013 | KR698353 | KR698340 | KR698327 | Genbank |
| Zhejiang | NB38-CHN-2013 | 2013 | KR698354 | KR698341 | KR698328 | Genbank |
| Zhejiang | NB39-CHN-2014 | 2014 | KR698355 | KR698342 | KR698329 | Genbank |
| Zhejiang | ZJZHSH-CXD-China-05-2012 | 2012 | KR017839 | KR017858 | KR017820 | Genbank |
| Zhejiang | ZJZHSH-FDE-China-06-2012 | 2012 | KR017840 | KR017859 | KR017821 | Genbank |
| Zhejiang | ZJZHSH-LHZH-China-06-2012 | 2012 | KR017838 | KR017857 | KR017819 | Genbank |
| Zhejiang | ZJZHSH-WRF-China-08-2014 | 2014 | KR017845 | KR017864 | KR017826 | Genbank |
| Zhejiang | ZJZHSH-YJX-China-06-2012 | 2012 | KR017841 | KR017860 | KR017822 | Genbank |
| Japan | SPL125A | N | AB983531 | AB985325 | AB985559 | Genbank |
| Japan | SPL057A | N | AB983500 | AB985295 | AB985526 | Genbank |
| Japan | SPL097A | N | AB983518 | AB985312 | AB985544 | Genbank |
| Japan | SPL112A | N | AB983525 | AB985319 | AB985551 | Genbank |
| Japan | SPL117A | N | AB983527 | AB985321 | AB985553 | Genbank |
| Japan | SPL120A | N | AB983528 | AB985322 | AB985556 | Genbank |
| Japan | SPL129A | N | AB983533 | AB985327 | AB985561 | Genbank |
| Henan | HNXY_188 | N | KC292328 | KC292274 | KC292301 | Genbank |
| Henan | HNXY_191 | N | KC292349 | KC292296 | KC292323 | Genbank |
| Henan | HNXY_202 | N | KC292351 | KC292325 | KC292298 | Genbank |
| Shandong | Shandong_SDLZtick12_2010 | 2012 | JQ684871 | JQ684872 | JQ684873 | Genbank |
